# VIEWING TIME AND FACIAL TRUSTWORTHINESS PERCEPTIONS: GIVE IT A SECOND THINK MAY NOT WORK FOR OLDER ADULTS

**DOI:** 10.1093/geroni/igz038.2887

**Published:** 2019-11-08

**Authors:** Yi Lu, Xu Ye, Yin Xiaowei, Zhang Xin

**Affiliations:** School of Psychological and Cognitive Sciences, Peking University, Beijing, Beijing, China (People’s Republic)

## Abstract

Older adults tend to rate higher on trustworthiness of unfamiliar faces than younger adults. Contrary to the notion that it would be beneficial for them to have enough time to process, cognitive control theory suggests that older adults’ facial trustworthiness evaluation increases over time, making them more vulnerable to fraud. The present study aimed at exploring age differences in trustworthiness perception of unfamiliar faces, and how viewing time could impact. A 2 (age: old vs. young) ×2 (viewing time: 500 ms vs. 3000 ms) ×2 (facial trustworthiness: control vs. low trustworthiness) factorial design was conducted. As expected a significant three-way interaction revealed that viewing time only influenced older adults’ facial trustworthiness evaluation, and only when given shorter viewing time did older adults exhibit accurate facial trustworthiness ratings as younger adults. These findings suggest that a second think in facial perception may not work for older adults.

